# Can Research Articles Published in Medical Journals be Used as Expert Evidence in Medical Negligence Cases?—A Cross-Sectional Retrospective Study of Indian Court Judgments

**DOI:** 10.1055/s-0045-1806761

**Published:** 2025-04-02

**Authors:** Aakash Sethi, Kalpita Shringarpure

**Affiliations:** 1 Department of Radiodiagnosis and Imaging, Postgraduate Institute of Medical Education and Research (PGIMER), Chandigarh, India; 2 Department of Preventive and Social Medicine, Government Medical College, Baroda, Gujarat, India

**Keywords:** malpractice, negligence, criminal law, medical ethics, standards of care, journal impact factor, practice guideline

## Abstract

**Background** The patient party is responsible for producing expert evidence to prove the negligence of a doctor, which becomes difficult due to lack of doctor's willingness to testify against other doctors. Impact factor (IF) is a surrogate to compare the quality of medical journals, which can be divided into low IF (< 10) and high IF (> 10). We aim to analyze various medical negligence cases where the medical journal was cited in court judgments on the parameters like court's verdict, IF of journals cited, compensation awarded, etc.

**Methods** This is a cross-sectional descriptive analysis. Judgments were accessed from www.scconline.com . IF was accessed from Clarivate Analytics 2019 ratings. Judgments having the word “Medical” AND “Negligence” in which either patient or doctor cited any journal data as evidence were included. The ci-square test was used as test of significance.

**Results** Twenty-six judgments met the inclusion criteria, with seven verdicts in favor of doctor (27%). The median IF was 2.455 with the *New England Journal of Medicine* having the highest IF (70.67). The median compensation awarded was 7.5 lakhs. The verdict of the court (doctor's win or loss) was not dependent on the IF (low IF or High IF) of the journal (chi-square = 0.16, *p* = 0.68).

**Conclusion** All types of courts handling medical negligence, viz., criminal court, consumer/civil court, writ court, and medical councils, accept medical journal research papers even as the sole evidence in the case of medical negligence. Most of the journals cited were low IF journals.

## Introduction

### Brief on Medical Negligence

Medical negligence is defined as “ *a breach of duty caused by the omission to do something which a reasonable man, would do, or doing something which a prudent and reasonable man would not do* .” 1

To prove the charges of medical negligence against a doctor, a patient has the burden to prove 2 :

1. Doctor's legal duty to exercise due care while treating the patient
2. Breach of that said legal duty (by showing the doctor did not follow the standard of care)
3. Damages were incurred by patient due to the said breach of duty

Under Indian laws, a doctor can be prosecuted under various statutes simultaneously, viz., civil law for damages/negligence (adjudicated by the civil courts), Consumer Protection Act 2019 (adjudicated by the district, state, and national consumer courts), criminal law (under Indian Penal Code, 1860, and tried by magistrates and appealed to Sessions and High Court), and national and state professional regulating bodies for misconduct under the 2002 Indian code of medical ethics. 3

The national consumer court is officially known as National Consumer Disputes Redressal Commission (NCDRC). Evidence-based guidelines set clinical standards, such that departure from them may require some explanation, but this departure does not constitute a de facto breach of the legal standard of care. 4 This stems from the precedent of the Bolam Hospital case. Bolam's test was adopted by the Supreme Court of India in the Jacob Mathew Case to test whether or not a doctor has acted negligently. 1 The Bolam's test says “ *A man need not possess the highest expert skill; it is well-established law that it is sufficient if he exercises the ordinary skill of an ordinary competent man exercising that particular art* .” The court in the same case in para 25 also held that mere deviation from the standard practice is not negligence.

### Role of Expert Evidence in Cases of Medical Negligence

The role of expert opinion given by the doctors is evident in view of the Bolam's test of “an *ordinary skill* and *competent man* .” The expert opinion becomes an important tool for the court to check whether or not the respondent doctor's conduct has fallen below the standard of care and are mandatory in some medical negligence cases. 5 However, a consumer advocacy group has said that patients claiming damages for medical negligence are often unable to prove their allegations because doctors are unwilling to testify against other doctors. 6

### Role of Journals in Medical Negligence

In the dearth of availability and will of doctors to testify against fellow professionals, as stated, the data cited in research articles pushed in journals might come in handy to prove the charges of negligence of the doctor. Nonetheless, the authenticity of the guidelines has to be established by having an expert testify that the guidelines are accurate representations of what they purport to be. 7

Even though journals have scientific and medico-legal importance, the average doctor spends not more than an hour a week on professional reading. 8 Thus, an average doctor might not be aware of all the latest evidence-based guidelines that have been published in journals unless they are regularly attending Continuing Medical Education, a mandate under the ethical standards published by India's regulating body of doctors. However, this section is hardly enforced in spirit.

The quality of a journal can be decided by measuring the impact factor (IF) of the journal. 9 Alternative bibliographies include Journal to Field Impact Score, SCImago Journal Rank, Source Normalized Impact per Paper, etc. 10

*Rationale* : Previous commentaries, 4 11 12 interview of U.S.-based negligence attorneys, 13 and judgments review of a Finland court 14 on the use of evidence-based guidelines and medical negligence were found after conducting a thorough literature search but such a study in the Indian context was lacking.

*Aim* : To analyze various medical negligence cases where the medical journal was cited as the source of evidence on the parameters of the type of case (criminal, civil, consumer dispute, etc.), compensation awarded by the court, nature of literature cited in court (journals, guidelines, etc.), the verdict of the court (claim of malpractice being rejected vs. accepted), the IF of the journals cited in negligence cases, and its association with the verdict of the court, if any. We hypothesize that courts accept articles published in medical journals as evidence in medical negligence cases.

## Materials and Methods

This is a descriptive retrospective record review of court judgments. The study duration was 6 weeks with 2 weeks for data collection, 2 weeks for analysis, and 2 weeks for article writing.

*Ethics statement* : We did not recruit any human participants, neither we collected nor stored any human data. We only used legal judgments (consumer court cases in our case), which were freely available online, as data source and hence ethical approval for institutional ethical committee was not sought. The Strengthening the Reporting of Observational Studies in Epidemiology checklist for cross-sectional study was followed

There were two primary sources of data collection. First, the court judgments like those of the High Court, Consumer Court, and Civil and Criminal courts, etc. were accessed from www.scconline.com . “ *Word search* ” feature was used on Supreme Court of India Cases (SCC) online and keywords like “ *medical* ,” “ *negligence* ,” “ *journal* ,” “ *article* ,” and “ *literature* ” were entered and judgments accessed. SCC online is one of the many online Indian legal search engines that contains judgments delivered by Indian courts. However, most if not all the legal search engines come with a disclaimer including SCC online that states that every effort is made to avoid any mistake or omission and the authenticity of this text must be verified from the original source. The Delhi Medical Council (DMC) disciplinary actions are accessed from their Web site. 15 The DMC verdicts and the NCDRC judgments were then screened using inclusion criteria. The inevitable limitation of the legal search engines is the authenticity of the judgments. The limitation of the DMC Web page is that not all judgments are uploaded on the Web site, with few of the complaints still under review. As the NCDRC and the DMC are judicial bodies, with judgments coming out each day, only judgments till specific dates can be studied. The DMC orders can be challenged by filing a petition with the Ethics and Medical Registration Board of the National Medical Commission (NMC). The orders of the NMC are not available online and hence cannot be studied to find if the DMC verdict has been upheld or overturned. The journal IF was accessed from Clarivate Analytics 2019 ratings by entering the journal name found from the relevant legal judgment analyzed. 16 All the variables were collected from these two sources. The journals were divided into two groups—high IF (> 10) and law IF, as per the National Institutes of Health classification. 17

The compensation awarded was converted in USD as per conversion rate on October 12, 2014. We used the consecutive purposive sampling method to arrive at study size and all judgments meeting the henceforth mentioned inclusion criteria and pronounced till the year 2020 were analyzed.

*Inclusion criteria* :

1. Judgments having the word “ *medical* ,” “ *negligence* ,” “ *journal* ,” “ *article* ,” and “ *literature* .”
2. Judgments with either patient or doctor referencing any journal data as evidence to prove their case.

*Bias* : The judgments were examined by the first and second authors separately, removing observer bias in the analysis of the qualitative characteristics (speciality of the doctor, surgery performed, presenting complaint, etc.). In cases of disagreement, a consensus was achieved. Consecutive sampling eliminated selection bias.

Data was entered in MS Excel and statistical tests were applied using the Web site “ *Social Statistics* .” 18 Qualitative data was expressed using percentages and quantitative data as median with interquartile range (IQR), which is the difference between 1st and the 3rd quartile. The chi-square test was used as a test of statistical significance for qualitative data. *p* -Values of < 0.05 were considered significant. The data sets generated during the current study are available from the corresponding author on reasonable request.

## Results

### Descriptive Data

**Fig. 1:**
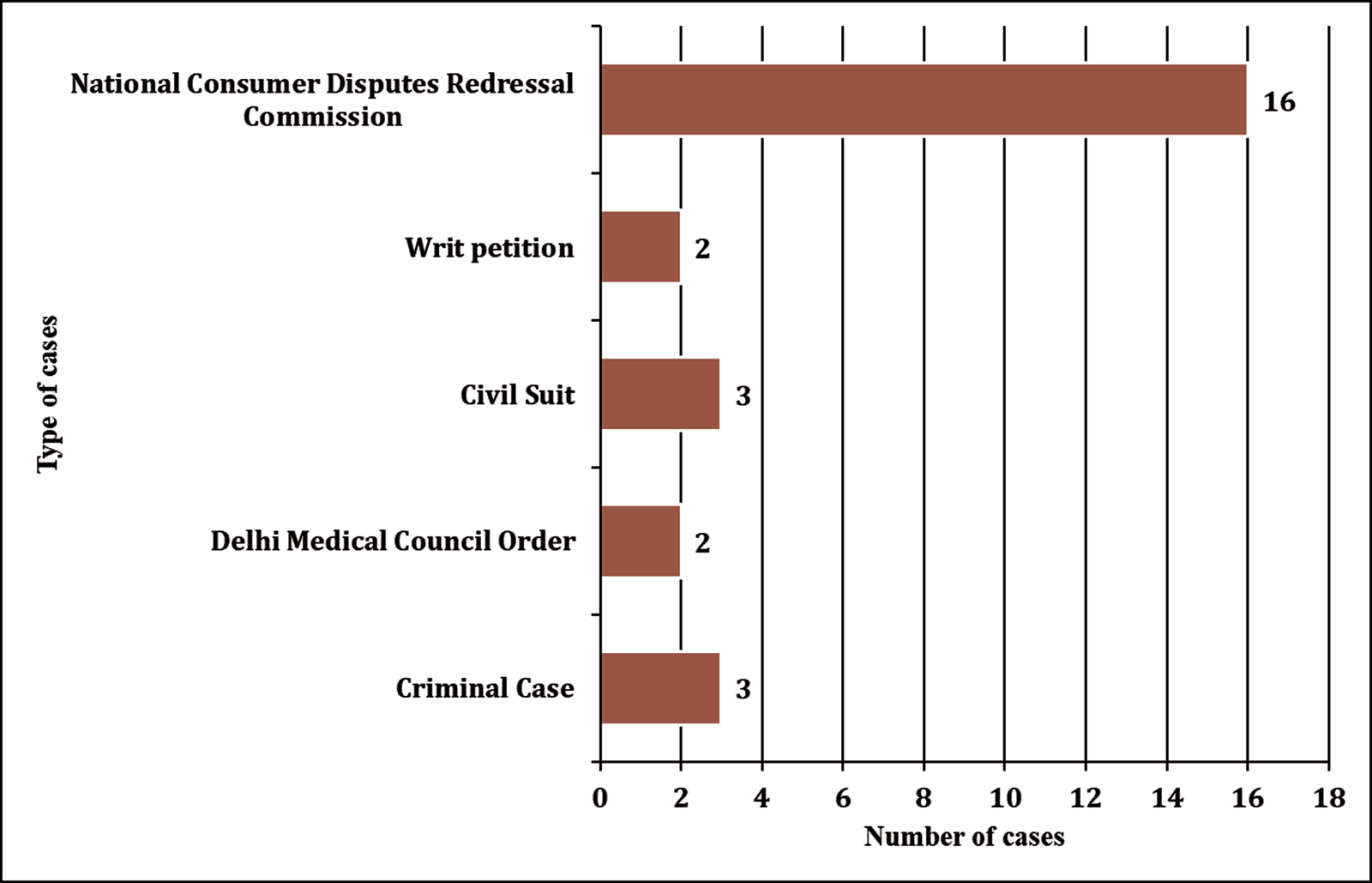
Judicial body/court where the case was filled ( *N* = 26).

**Fig. 2:**
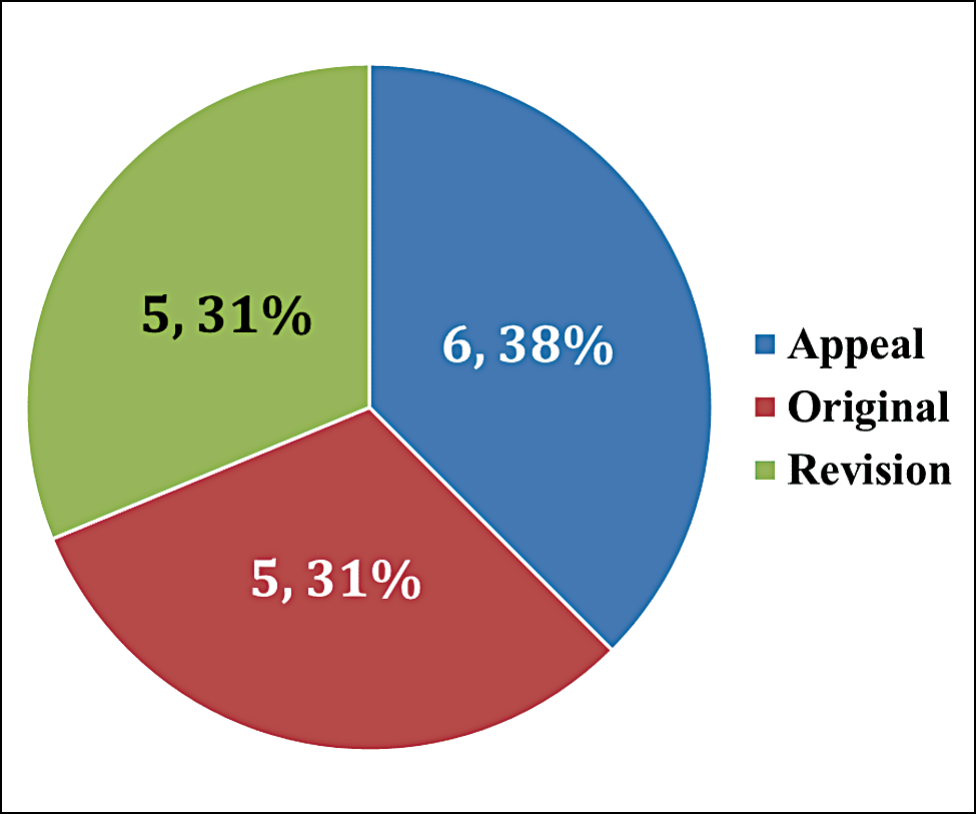
Division of cases filled in the National Consumer Dispute Redressal Commission ( *N* = 16).

Twenty-six judgments met the inclusion criteria. There were 16 (62%) Consumer Court cases, 3 cases each of civil lawsuits and criminal cases, and 2 cases each of writ petitions and DMC orders ( Fig. 1 ). Out of the 16 cases of NCDRC, the appeals against lower court judgments were the most common type of judgments found ( Fig. 2 ). In seven (27%) cases, the verdict went in favor of the doctor. Surgeons ( *n* = 14, 53.8%) were more frequently implicated than their physician ( *n* = 12 45.8%) counterparts. There was a single case against a pathologist, rest among surgical and medical specialties. Among the surgeons, the gynecologists, and the physician counterparts, internal medicine specialists were the most commonly implicated specialists ( Tables 1 and 2 ).

**Table 1:** Surgical and allied specialties ( *n* = 14)

Gynecologist	7 (50%)
Ophthalmologist	2
Orthopedic	2
ENT	1
Surgical oncologist	1
General surgeons	1

Abbreviation: ENT, ear, nose, and throat.

**Table 2:** Medicine and allied specialties ( *n* = 11)

Internal medicine	5
Radiologist	3
Cardiologist	2
Gastroenterologist	1

### Quantitative Data

The median compensation awarded by the court was US$ 8,923 (IQR = US$ 2,909 to US$ 27,366).

### Main Results

**Fig. 3:**
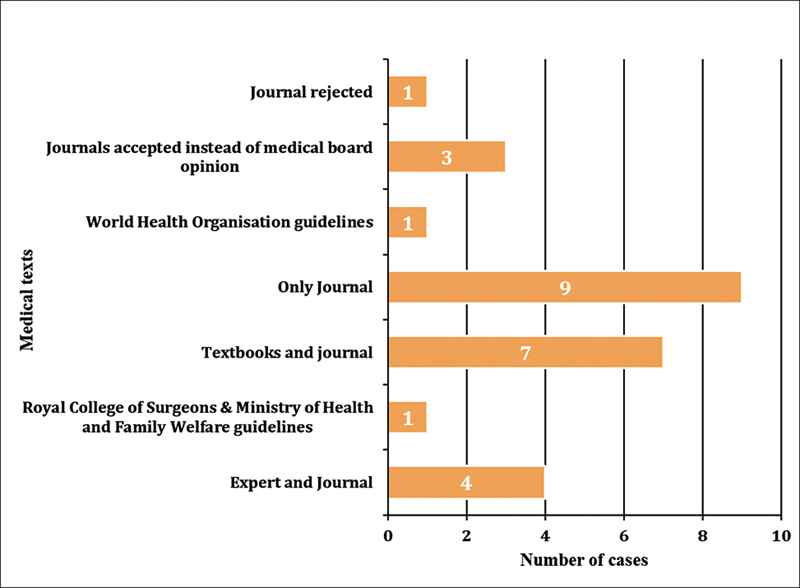
Type of medical texts that were accepted by the court during its hearing ( *N* = 26).

The court relied on various sources including guidelines by the Ministry of Health and Family Welfare (MOHFW), the Royal College of Surgeons, the World Health Organization (WHO), and even textbooks apart from standalone articles. The court also considered researches published in journals as the only evidence (9 cases, 34.6%). It even rejected the medical board's opinion (3 cases, 11.5%) and relied solely on the evidence published in journals ( Fig. 3 ). The highest IF was of the *New England Journal of Medicine* (IF = 70.670). The lowest IF cited was 0.871 ( *Southern Medical Journal* ). The wide variation in the IF is to be noted. IF was unavailable on Clarivate Analytics for 13 journals and were not accounted for calculation of median. The median IF was 2.455 (IQR = 1.99–5.40). Using the chi-square test we found that the citation of high IF journals (IF > 10) had no bearing on the outcome of the case (doctor winning vs. losing) (chi-square = 0.16. *p* = 0.68).

## Discussion

The present study highlights the importance of journals from a medico-legal perspective, a shift from the traditional academic purpose they served and adds to evidence laid down by the few studies that studied the role of clinical practice guidelines (CPGs) in medical negligence cases. The reliance of courts on journals and published data (arguably a form of written expert opinion) and not just verbal expert opinion warrants physicians to keep themselves updated. Indian jurisprudence on medical negligence has recently matured and booming (in contrast to British and American courts), thus a study of how courts in India treat the data cited in journals was needed and hence was undertaken. The key implication of our findings is the discovery that all types of courts that can hold doctors guilty of breaking law, accept expert evidence in the form of journal articles. The strength of our study is that it has provided statistical analysis (as discussed further) alongside reviewing the text of the judgment for court findings.

Limitations of our study include unavailability of IF for 13 journals that were cited in the court judgment, which highlights the inherent problem with the authenticity of the type of data that was cited, as discussed later. Second, as older IF could not be found, the 2019 data was used. The judgments were as old as 2004 and hence current IF could vary from the one previously reported.

Data cited in the journal represent an alternate and more recently updated source of credible medical information than textbooks. In studies conducted on use of CPGs, it was found that those are likely to be used by patients in 54% of cases and by doctors in 22.7% of negligence suits out of 578 surveyed doctors. This study also analyzed 259 insurance company claims in the United States and found 17 claims where CPG were used. Out of these 17 cases, only one case resulted in jury ruling in favor of the doctor (∼6%), much less than those found in our study. 13 The maximum cases involved claims against obstetricians (11 out of 17), similar to our findings. Analysis by Carter et al showed 3 out of 15 cases (20%) of nonconsecutive clinical negligence claims in one large NHS Trust. We had two cases where guidelines by professional bodies were followed (viz., Royal College of Surgeons, Indian MOHFW, and the WHO). 3 A total of 110 lawyers completed a 14-question survey, where they claimed that CPGs were used by them as evidence in 89% of the cases they argued for.

The nature of literature cited in court (journals, guidelines, etc.) included both CPG by expert societies (consensus statements) and individual research articles in journals. In three cases, the opinion of the medical board was rejected while relying on journals like the *British Medical Journal* (BMJ) and the *Journal of the Royal Society of Medicine* . The NCDRC in the case of Dr. Indu Sharma said “ *…We reject the expert opinion….as they* *were from the same hospital and more chances of interested witnesses* .” 19 Thus, journal data represents an unbiased form of “expert opinion” and are a more reliable source of evidence.

In the case of Prof. Dipak Ghosh the state consumer court said “ *Our utmost admiration towards* *the aforesaid valuable opinion of the expert committee* *notwithstanding, we have not allowed our consciousness to be completely swayed away by such findings.* *Rather, in the interests of natural justice, we have referred to various medical journals, write-ups, and research findings* *and on due consideration of the same, reached the following conclusion* ” (emphasis supplied). 20

On breach of fundamental rights, a writ petition can be filed under the Indian Constitution Article 32 (in the Supreme Court) and Article 226 (in the High Court) claiming compensation. 21 Even in the writ petition the courts relied on evidence published in journals, which is a relatively less frequent means of claiming compensation for medical negligence. The Gauhati High Court decided in favor of the doctor in case of failed sterilization. It relied on national and international published data to prove that sterilization had its limitations and thus the doctor cannot be held negligent only based on the failure of sterilization in view of standard laid down in the *Bolam's test* . 22

The DMC while deciding on a case alleging negligence during preparation of second trimester antenatal ultrasound report, held the doctor not negligent, relied on notable journals like the *Ultrasound in Obstetrics & Gynecology and Pediatrics* . It noted that “ *left hypoplastic heart syndrome may be missed on antenatal ultrasounds in 30-40 percent cases as per* *existing medical literature* .” 23

Apart from showing they are relevant to the present case, the patient/doctor must also show that the body promulgating the guidelines is a “ *well-respected medical authority* ” and that the process through which the guidelines were “ *developed and updated was sound* .” 24 This is a result of a jurisprudential shift in standards applied in medical negligence cases from the *Bolam Standard* to the *Bolitho Standards.* The Bolam's test, says the following “ *…. a person is not liable in negligence because someone else of greater skill and knowledge would have prescribed different treatment or operated differently; nor is he guilty of negligence if he has acted in accordance to a practice accepted as proper by* *a responsible body of medical men skilled in that particular art* , *even though a body of adverse opinion also existed among medical men* ” (emphasis supplied). 25

Later, in the Bolitho case where Bolam's standards were overruled, 26 Lord Browne Wilkinson delivering the judgment said “ *The court has to be satisfied that the exponents of the body of opinion relied upon can demonstrate that such opinion has a logical basis….the experts have directed their minds to the question of comparative risks and benefits and have reached a defensible conclusion on the matter* .” Thus, according to Bolitho's test, production of favorable expert opinion is not a valid defence, the opinion must also be logically valid for it to be accepted by the courts.

Although the Indian judiciary has not accepted the Bolitho standards as the norm when the shift happens, it could result in the doctor being held guilty of negligence even when he had followed the expert guidelines if such opinions do not have a “logical basis.” 27

The issue of the soundness of guidelines and the credibility of the journal article was never in question in any of the verdicts we analyzed. Objectively, this is reflected by our statistical analysis. We found that the IF of the journals cited did not show any significant relation with the verdict of the court. This is important because authors have often questioned these guidelines on the criteria of credibility. An analysis published in the *Lancet* shows that out of 431 clinical guidelines, produced by specialty societies between January and July 1988, 67% did not report any description of the type of stakeholders involved in guideline development or use, and 82% provided no explicit grading of the strength of recommendations. 28

More recently, a study published in the *BMJ* where 626 consensus statements and another where 127 Norwegian-based guidelines were evaluated using the Appraisal of Guidelines Research and Evaluation (AGREE) instrument showed striking results. The Norwegian study concluded that most clinical guidelines do not fulfill the quality criteria 29 and the one published in the *BMJ* study concluded that despite some increase in the quality of CPGs over time, the quality scores as measured have remained moderate to low. 30 This suggests that simply accepting a consensus statement from a medical body is insufficient, and that deviating from the basic guidelines should be encouraged.

There are, however, limitations in using CPG and journal articles in cases of medical negligence. CPGs are not often translated to practice due to factors like complexity and different target groups tested while formulating the guidelines. The applicability of the guidelines is limited to resource-poor countries as most if not all the CPGs are formulated in resource-sufficient countries. For example, payment and cost issues are the most cited obstacles to guidelines implementation. 31 Journal articles may not fully capture the complex contextual factors that contribute to medical negligence, such as systemic or environmental factors. 32 Journals may be more likely to publish articles with positive or significant findings, rather than negative or inconclusive results, which means there is inherent publication bias present. 33 Generally speaking, journal articles cover broad concepts and research study results. They might not deal with the particulars of a given clinical instance. Individuals may not benefit from what is beneficial to the population as a whole. Therefore, less tailored treatments for individuals with particular requirements might suffer from the widely cited advantage of professional guidelines—more consistent practice patterns and less variety. 7 34 Given there are flaws with journal articles themselves can make their admissibility in court of law a question.

In the *Malay Kumar Ganguly case* 35 and its connected civil negligence matter *Balram Prasad Versus Kunal Saha* , 36 the Supreme Court relied on textbooks and publications like the *Journal of Association of Physicians of India* , Goodman and Gillman, Harrison's Principle of Internal Medicine, among others. Concurring with the trial court opinion, it found that the treating doctor did not follow the treatment guidelines provided for in the journals and awarded the maximum compensation in the history of medical malpractice claims in India, a whopping 6.08 crore rupees.

A study by Yadav and Rastogi 37 showed that about 60% of cases of medical negligence in Delhi were against surgery specialists. Our study revealed similar results mainly owing to the risks involved in invasive surgical approach, providing more instances of alleged error on the treating doctor.

The IF has its own set of pros and cons. It is simple to calculate and allows for interdisciplinary (e.g., medicine, physics, astronomy) comparison of journals. Its limitations or drawbacks include it being an arithmetic mean of total citations done and hence does not account for skewed citation distribution. It also promotes self-citation and publication of review articles. The citation window of 2 years taken as a basis for the calculations is too short to judge the impact of publications and hence limits the utility of the IF.

Future research studies could focus on not only IF but also include data from other available journal statistics as described previously. There are implications for the judicial as well as clinical training of all stakeholders involved—the patient, the expert witness, the treating physician, policy makers, and the professional bodies making the guidelines. The terminology used in court makes a difference for the expert witness. A word like “it is probable” will turn a case in the party's favor and demonstrate a confidence level of more than 50%, whereas a term like “it is possible” indicates a confidence level of less than 50%. Experts should use terms like “consistent with” or “inconsistent with” more often. 38 Establishing the standard of care in medical disputes through the use of well-written and authoritative clinical standards has several advantages. When used wisely, they could improve health care quality and give physicians more clarity on what the law requires of them. 7 With regards to the professional bodies and policy makers, it is crucial to stop subpar guidelines from making to the final stop of publication as they can be influencing legal standards. For the patient, the guidelines serve as helpful in proving medical negligence, when most of the medical professionals might be unwilling to testify. As the previous data has shown, the guidelines are more commonly used for inculpatory purpose (against the doctor) then otherwise. 4 13

## Conclusion

Our results show that all types of courts handling medical negligence, viz., criminal court, consumer court, civil court, and writ court, accept medical journal research papers even as sole evidence in the case. Both the patient and doctor can produce the evidence in the form of research articles or practice guidelines. Thus, the hypothesis is accepted. Summary table is represented as Table 3 .

**Table 3:** Summary table of outcomes

Parameter	Details
Total judgments analyzed	26
Types of cases	• Consumer court: 16 cases (62%) • Civil lawsuits: 3 cases (12%) • Criminal cases: 3 cases (12%) • Writ petitions: 2 cases (8%) • Delhi Medical Council orders: 2 cases (8%)
Verdicts in favor of doctors	7 cases (27%)
Median compensation awarded	7.5 lakhs (approx. USD $8,923)
Median impact factor (IF)	2.455 (IQR: 1.99–5.40)
Highest impact factor cited	70.67 ( *New England Journal of Medicine* )
Lowest impact factor cited	0.871 ( *Southern Medical Journal* )
Significance of impact factor	No significant relation between journal IF and case outcome (chi-square = 0.16, *p* = 0.68)
Evidence sources accepted	• Journals used as sole evidence in 9 cases (34.6%) • Other sources: guidelines, textbooks, medical board opinions
Outcomes for journal evidence	Courts accepted journal data as evidence, regardless of IF, but authenticity was not questioned

Abbreviation: IQR, interquartile range.

Given its importance in medico-legal cases, doctors should be trained early on in their careers to actively read standard journals to improve and update their knowledge.

Journal editorial team should increase their scrutiny (via peer review process, action against authors including blacklisting for violation of ethical breach) because an article claiming a certain positive finding (e.g., the success of a surgical procedure to be favored during publication) can lead to adverse judgment by the court.
